# Reemergence of Japanese Encephalitis in South Korea, 2010–2015

**DOI:** 10.3201/eid2210.160288

**Published:** 2016-10

**Authors:** Jun-Sang Sunwoo, Keun-Hwa Jung, Soon-Tae Lee, Sang Kun Lee, Kon Chu

**Affiliations:** Seoul National University Hospital, Seoul, South Korea

**Keywords:** Japanese encephalitis, South Korea, vaccination, viruses, arbovirus, *Culex tritaeniorhynchus*, reemerging infectious diseases, mosquitoes, vector-borne infections

**To the Editor:** Japanese encephalitis (JE) is caused by a virus transmitted by *Culex tritaeniorhynchus* mosquitoes. JE was the major public health concern in South Korea until the late 1960s, with several thousand cases reported annually. The national vaccination program with the inactivated mouse brain–derived Nakayama strain was initiated in 1983 and targeted children <15 years of age. During 1983–2000, annual booster vaccinations were given to children <15 years of age, but in 2000, the booster schedule was changed to 2 doses (1 dose each) for children 6 and 12 years of age. The live attenuated JE vaccine SA 14-14-2 was introduced in 2002 and included in the national immunization program in 2014. After introduction of the mandatory immunization program, JE was nearly eliminated; during most of the past 3 decades, <0.02 cases per 100,000 population have been reported annually (*1*). However, since 2010, JE has reemerged in South Korea. We describe epidemiologic data for JE, focusing on the recent increase in number of cases in South Korea. We accessed demographic information from the disease web statistics system provided by the Korea Centers for Disease Control and Prevention (*2*). Our study was exempted from review by the Institutional Review Board at Seoul National University Hospital (E-1602-053-739).

During 2010–2015, South Korea reported 129 JE cases (*2*). JE was diagnosed on the basis of clinical signs and symptoms and laboratory examination that showed either the presence of JE virus (JEV)–specific IgM in serum or cerebrospinal fluid samples or identification of a >4-fold increase in neutralizing antibody titers between the acute and convalescent stages. Laboratory procedures were conducted by the Korea Centers for Disease Control and Prevention, as described (*3*). Clinical features suggesting JEV infection were acute encephalitis syndrome (defined as altered consciousness with fever or seizures) and focal neurologic deficits. Reports excluded clinically suspected but serologically unconfirmed cases. Among the 129 confirmed cases, only 1 (0.78%) case-patient had documented evidence of JEV vaccination. Domestic or international travel history was evident in 18 (14.0%) case-patients; 16 (12.4%) were found to live in proximity to a pigsty; and 8 (6.2%) were foreign-born residents.

Annual incidences of JE have increased markedly since 2010, except for 2011, when only 3 cases were reported (Figure, panel A). Incidence was highest during 2015, when 40 cases were reported. A total of 19 patients died during 2010–2014 (overall case-fatality rate 21.3%), whereas during the previous 25 years (1985–2009), only 5 deaths were attributable to JE (*4*). 

**Figure:**
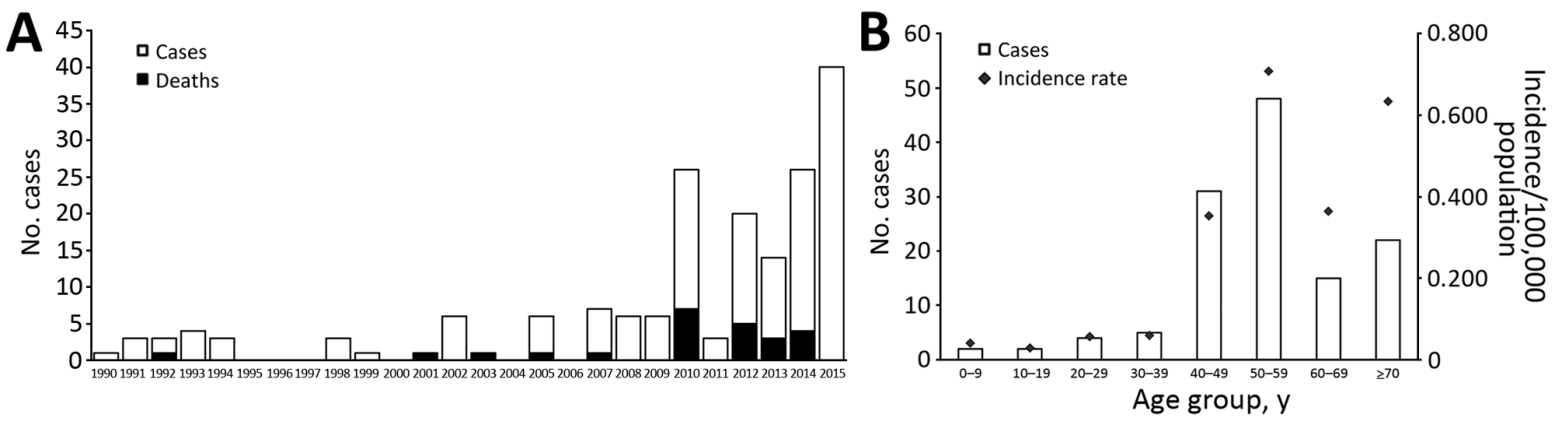
Reemergence of Japanese encephalitis in South Korea. A) Number of cases and deaths caused by Japanese encephalitis by year, 1990–2015. Number of deaths for 2015 is not shown because final data were unavailable. B) Number of cases and incidence of Japanese encephalitis by age group, 2010–2015. Population data for the denominator originated from the future population projection data of the Korea Statistical Information Service through 1992 and from the mid-year population data from the resident registration system as of 1993. All disease data shown in the figure were provided by the Korea Centers for Disease Control and Prevention, Infectious Disease Statistics System (http://is.cdc.go.kr/dstat/index.jsp).

Median age of the 129 patients with JE was 53 years (interquartile range 46.5–62.0). Most (73 [56.6%]) patients were male; 56 (43.4%) were female. On average, affected female patients were older than male patients (mean 56.6 ± 16.3 years vs. 51.4 ± 12.8 years; p = 0.017 by Mann-Whitney U test). When patients were stratified by age, those 50–59 years of age (37.2%) were the most affected group, followed by those 40–49 years of age (24%). Patients <19 years of age accounted for only 3.1% of cases (Figure, panel B). Analysis of the monthly incidence of JE revealed a distinctive summer peak; 109 (84.5%) cases occurred during August–October, suggesting a temporal association with activity of mosquito vectors (*5*). Analysis of geographic distribution showed that 58 (45%) cases originated in Seoul, the capital of South Korea, or in Gyeonggi Province, the area surrounding Seoul (Technical Appendix Figure).

Our findings indicated that reemerging JE predominantly affects unvaccinated adults >40 years of age. Shifts in age distribution toward older groups after initiation of vaccination programs were also evident in Japan and Taiwan (*6*,*7*). Many researchers believe that prolonged periods with near elimination of JE over the past 3 decades and an unvaccinated adult population have contributed to older adults’ high vulnerability to JE. However, recent JEV seroprevalence data showed that 98.1% of persons in high-risk age groups had neutralizing antibodies, with no differences appearing among age groups (*8*). Although those findings conflict with the assumption that lack of vaccination among older adults contributes to vulnerability to infection, high seroprevalences could be explained by natural infection resulting from the large epidemics of the 1950s and 1960s.

Our study has several limitations. For example, information on clinical features and outcomes of patients, except for death, was unavailable, and we could not determine prognostic factors for recent JE cases. Because details of each patient’s travel history was not identified, we could not clearly understand the mechanism of JEV transmission. In addition, we do not explore the possible cause of JE reemergence. Moreover, although JE incidence was detected by the national surveillance system, incidence might be underestimated because the database identified only serologically confirmed cases.

JE vaccination is presumed to have failed to induce lifelong immunity so that older age groups become susceptible again. Further research is warranted to determine the long-term protection against JEV after primary vaccination. Moreover, future studies should address the need for booster vaccination for adults to maintain immunity against JEV.

Technical Appendix Geographic distribution of Japanese encephalitis cases in South Korea, 2010–2015.
