# Supplementary material for: Reemergence of Japanese Encephalitis in South Korea, 2010–2015
Source: Emerg Infect Dis. 2016 Oct;22(10):1841–3. doi: 10.3201/eid2210.160288 (PMC5038436; doi:10.3201/eid2210.160288)
Supplement: Technical Appendix — Geographic distribution of Japanese encephalitis cases in South Korea, 2010–2015. [file 16-0288-Techapp-s1.pdf]

Article DOI: http://dx.doi.org/10.3201/eid2210.160288

Reemergence of Japanese Encephalitis in South Korea, 2010–2015

Technical Appendix

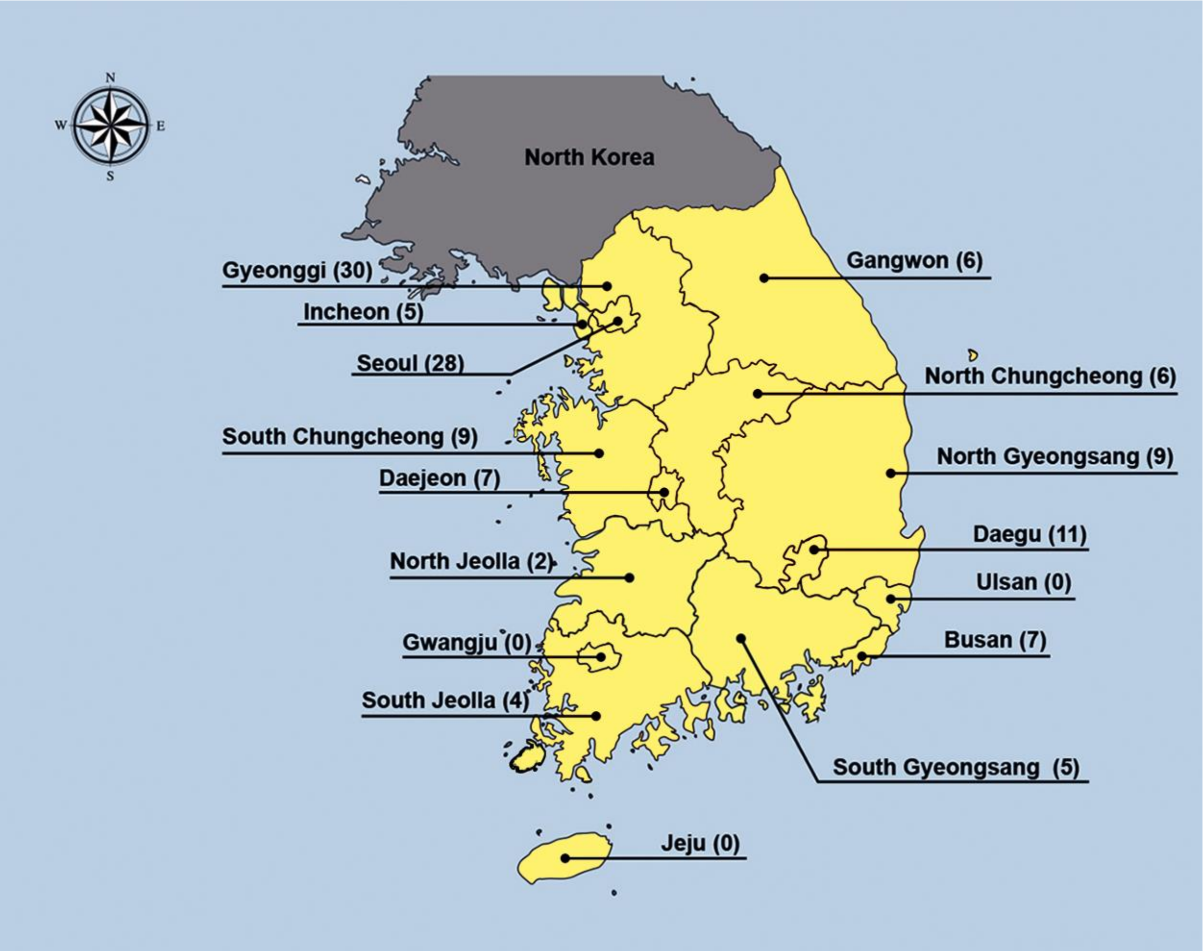

Technical Appendix Figure. Geographic distribution of Japanese encephalitis cases in South Korea, 2010–2015. Numbers in parentheses indicate number of Japanese encephalitis cases reported for each area. All data were provided by the Korea Centers for Disease Control and Prevention, Infectious Disease Statistics System (http://is.cdc.go.kr/dstat/index.jsp)
